# Silicone oil tamponade in vitreoretinal surgery and future perspective: a narrative review

**DOI:** 10.1007/s00417-026-07301-5

**Published:** 2026-06-05

**Authors:** Cesare Mariotti, Alessio Muzi, Giulia Gregori, Oscar Matteo Gagliardi, Danilo Iannetta, Veronica Mogetta, Alberto Quarta, Maria Ludovica Ruggeri, Lorenzo Mangoni, Clara Rizzo, Rodolfo Mastropasqua, Marco Lupidi

**Affiliations:** 1 Department of Experimental and Clinical Medicine, Eye Clinic, Polytechnic University of Marche, Ancona, Italy; 2 Department of Ophthalmology, Humanitas Gradenigo, Turin, Italy; 3 Department of Sense Organs, Sapienza University of Rome, Rome, Italy; 4 Department of Neuroscience, Imaging and Clinical Sciences, University G. D’Annunzio Chieti-Pescara, Chieti, Italy; 5 Di.N.O.G.Mi, Fondazione Italiana Macula ETS, University Eye Clinic, Genova, Italy; 6 Department of Neurosciences, Psychology, Drug Research, and Child Health, Eye Clinic, University of Florence, AOU Careggi, Florence, Italy

**Keywords:** Silicone Oil, Tamponade agent, Hydrogel, Retinal detachment, Endophthalmitis

## Abstract

**Purpose:**

Silicone oil (SO) represents an essential ocular tamponade in complex vitreoretinal surgery, due to its transparency, chemical inertness, and long-term stability. This review aims to describe the current role of SO in vitreoretinal surgery, highlighting its indications, physicochemical characteristics, and the spectrum of associated complications. Special attention is given to the timing of removal and the balance between efficacy and safety.

**Methods:**

We performed a narrative review of PubMed, Scopus and Google Scholar, focusing on indications, physicochemical determinants, complications and removal strategies of SO. The literature search was conducted up to January 2026. Inclusion was limited to English-language clinical and translational studies.

**Results:**

Silicone oil provides a reliable long-term internal tamponade and contributes to favorable anatomical outcomes in eyes at high risk of recurrent retinal detachment. However, complications such as emulsification, ocular hypertension, corneal decompensation, cataract formation, and unexplained visual loss remain significant concerns. The risk of these adverse events increases with the duration of tamponade, underscoring the need for timely removal. Many centers plan removal around 3–6 months, tailored to retinal stability, IOP, and anterior SO migration. In selected cases with particularly severe or recurrent pathology, extended tamponade can still offer functional benefits and preserve useful vision.

**Conclusion:**

Silicone oil continues to play a central role in complex vitreoretinal surgery. Careful patient selection, appropriate choice of oil formulation, meticulous surgical technique, and vigilant follow-up are critical to optimizing outcomes. Future directions should focus on the development of improved SO formulations and surgical strategies to enhance safety, minimize complications, and expand therapeutic indications.

## Key Messages

*****What is known***:**

- Silicone oil (SO) is the long-term endotamponade for complex retinal detachment, with outcomes driven by indication, surgicalquality, and timely removal.
- Physicochemical properties (viscosity, density, interfacial behavior) should guide selection among standard and heavy SO; prolongeddwell time increases emulsification-related risks.

*****What is new***:**

- This review integrates physicochemical, clinical, and surgical evidence to support a decision-based approach to silicone oil selectionand timing of removal in complex vitreoretinal surgery.
- Upcoming EU regulatory restrictions on PolyFluoroAlkyl Substances–related (PFAS-related) compounds may impact heavy siliconeoils (HSOs) containing fluorinated components, while conventional PDMS-based SOs remain unaffected; ongoing monitoring will becrucial for clinical and industrial adaptation.

## Introduction

In vitreoretinal surgery, a tamponade serves as a vitreous substitute that maintains adhesion between the retina and the retinal pigment epithelium for the required duration, thereby restoring the original volume of the vitreous cavity.

Silicone oil (SO) is a long-term tamponade introduced in 1962 for the management of retinal detachment (RD) [1], and it remains the preferred option in complex vitreoretinal surgical cases. SO is particularly suitable for intraocular use owing to its transparency, chemical inertness, viscosity, and buoyancy over the residual vitreous. While these properties are also shared by intraocular gases, gas tamponades are characterized by a progressive reduction in volume and, consequently, a limited duration of action. Therefore, they may not provide a sufficiently prolonged tamponade effect in complex vitreoretinal conditions. In contrast, SO provides a stable and sustained tamponade that persists until surgical removal, making it more suitable for cases requiring long-term retinal support. SO is considered an effective tamponade because its physicochemical properties reduce fluid ingress through retinal breaks and help maintain reattachment, with minimal subretinal migration. Initially, impurities in the manufacturing process limited its clinical application and delayed approval from the Food and Drug Administration until 1994 [2]. Subsequently, the development of highly purified SOs improved their safety profile [2, 3]. Pathological pictures in which the use of SO is indicated include complex vitreoretinal surgeries, such as RD associated with vitreoretinal proliferation (PVR), multiple recurrent RD, giant retinal tears, ocular trauma, and proliferative diabetic retinopathy (PDR). Additional indications include cases in which postoperative positioning cannot be adequately maintained or in patients residing at high altitudes. The tamponade role must be temporary, as it complements the retinopexy in achieving and stabilizing retinal reattachment. Then, over time, SOs must be removed, since SO intraocular retention is associated with time-related complications, mostly resulting from emulsification phenomena, particularly when using low-viscosity oil [3].

The purpose of this narrative review is to integrate molecular and clinical evidence regarding SO tamponade, highlighting physicochemical determinants of efficacy and emulsification-related toxicity and guiding tamponade selection. We also outline future directions, covering emerging substitutes and regulatory considerations with clinical impact.

## Methodology

**Fig. 1:**
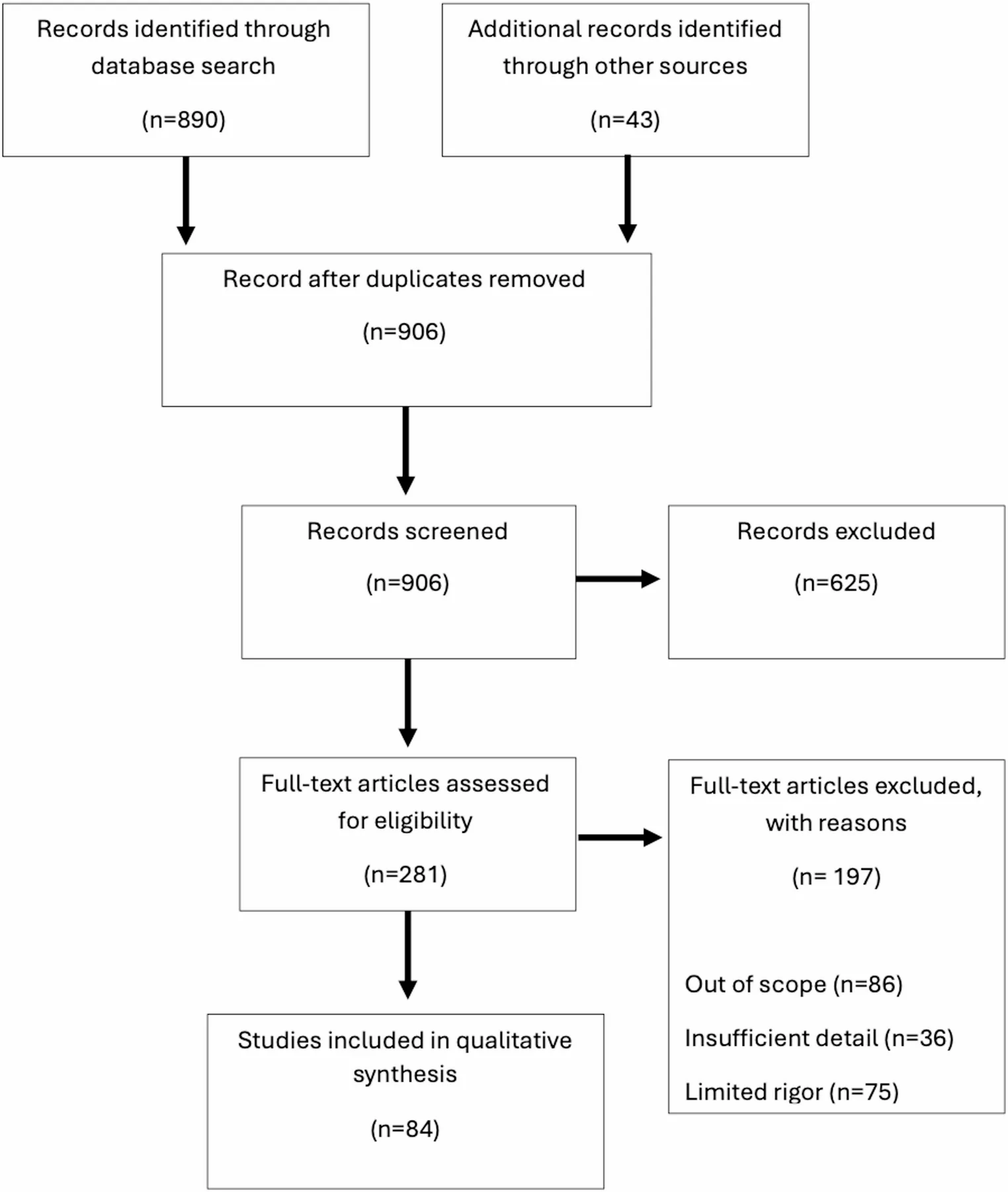
Flow diagram of the literature search and study selection process

We performed a narrative literature review using PubMed/MEDLINE, Scopus, and Google Scholar. The search was updated in January 2026. The main search terms were: silicone oil, heavy silicone oil, polydimethylsiloxane (PDMS), vitreoretinal surgery, pars plana vitrectomy, retinal detachment, proliferative vitreoretinopathy, tamponade, emulsification, complications, and silicone oil removal. We prioritized English-language clinical and translational studies published between January 1, 2000, through December 31 2025 and we also included a limited number of earlier landmark papers when needed for historical background. Additional relevant articles were identified by screening reference lists and forward citations. The core Boolean strings were adapted to each database. A flow diagram summarizing the selection process is provided (Fig. 1).

## Results

### Physicochemical properties of silicon oil

The selection of a tamponade agent should be guided by its physicochemical properties, which may vary across different silicone oil formulations, each serving a specific purpose according to the clinical context.

**Fig. 2:**
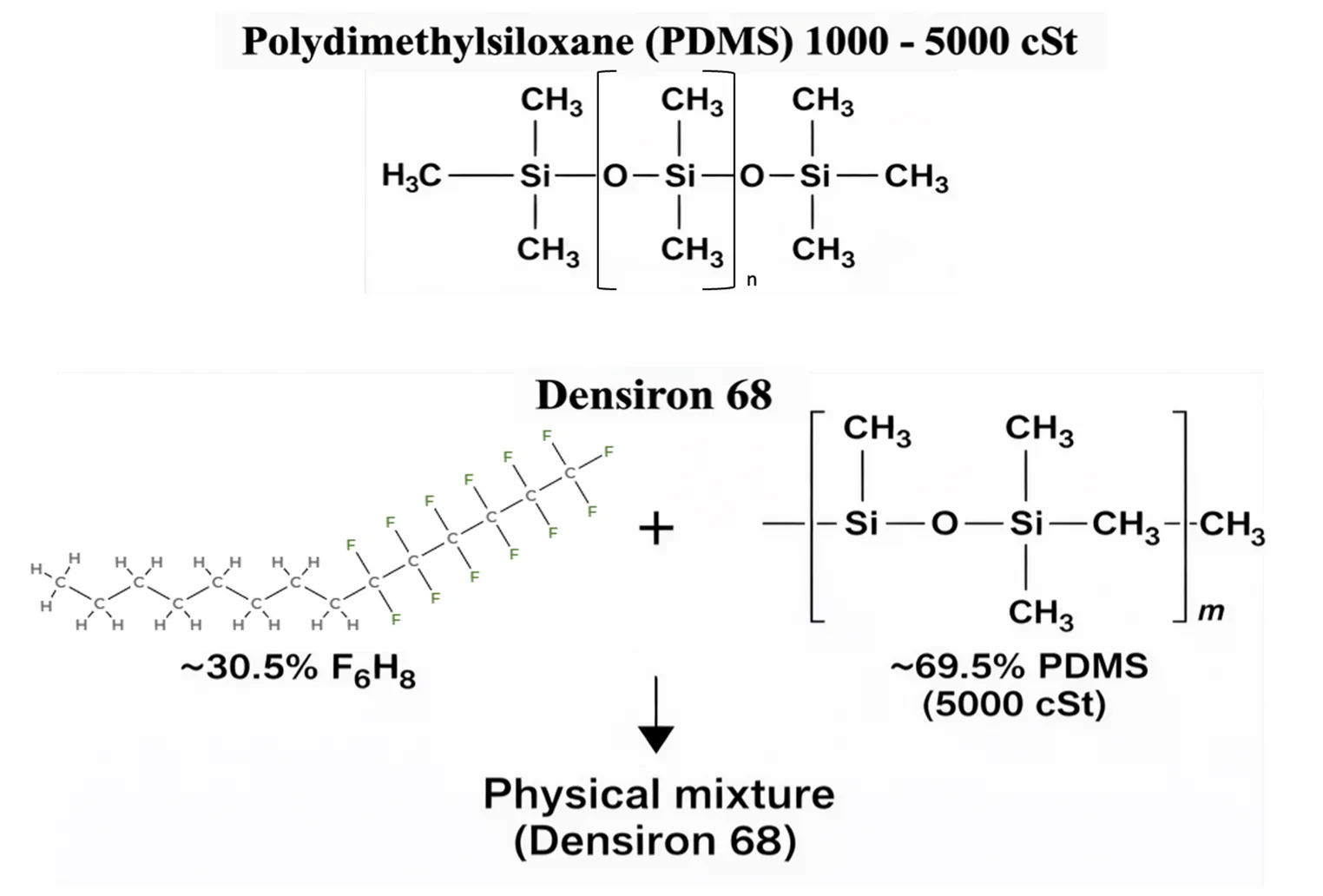
Chemical structures of ophthalmic silicone-based tamponade agents

Chemically, SO is a polydimethylsiloxane (PDMS), a linear polymer composed of chains water-insoluble siloxane (-Si-O). units with methyl side groups (-CH₃) bound to the silicon atoms. These methyl groups can be partially substituted by other radicals (e.g., phenyl, vinyl, or trifluoropropyl) to modify the physicochemical behavior of the oil. (Table 1) Readers are referred to Fig. 2 for a schematic representation of PDMS structure and commonly used modifications. The molecular weight of PDMS chains (typically 37–65 kDa) directly determines viscosity, with higher molecular weight corresponding to higher viscosity (e.g., ~ 1000 cSt vs. ~ 5000 cSt). These chemical features govern all clinically relevant physical properties, including density, viscosity, and interfacial behavior [4].

**Table 1:** Main silicone oils used in ophthalmology and their physicochemical properties

Silicone Oil (SO)	Type	Viscosity (cSt)	Specific Gravity	Refractive Index	Surface Tension (mN/m)	Main Clinical Notes
**Silicone oil 1000 cSt**	Standard SO	1000	~ 0.97	~ 1.404	~ 35	Widely used; relatively easy to inject/remove; higher risk of emulsification compared to higher viscosities.
**Silicone oil 2000 cSt**	Standard SO	2000	~ 0.97	~ 1.404	~ 35	Increased stability vs. 1000 cSt; still relatively manageable for removal; commonly used.
**Silicone oil 5000 cSt**	Standard SO	5000	~ 0.97	~ 1.404	~ 35	Lower emulsification risk; technically more difficult to inject/remove due to viscosity.
**Oxane 5700** (Bausch & Lomb)	Heavy SO	~ 5700	1.02	~ 1.404	~ 35	Mixture of SO and semifluorinated alkanes; heavier than water; indicated for inferior retinal breaks or PVR.
**Densiron 68** (Fluoron)	Heavy SO	~ 1400–1500	1.06	~ 1.404	~ 40	Blend of SO 5000 cSt + perfluorohexyloctane; heavier than water; effective for inferior RDs; moderate removal difficulty.
**Densiron Xtra** (Fluoron)	Heavy SO	~ 1200–1300	~ 1.06	~ 1.404	~ 40	Modified formulation of Densiron 68 with increased viscosity and improved resistance to emulsification; used for inferior retinal detachments and complex PVR cases.
**HWS 46-3000** (Heavy Water-Silicone blend)	Heavy SO	~ 3000	1.03–1.04	~ 1.404	~ 38	Alternative heavy tamponade; limited clinical diffusion.
**Oxane HD** (Bausch & Lomb)	Heavy SO	~ 3300	1.02	~ 1.404	~ 35–38	Blend of SO and RMN3 (perfluorohexyloctane derivative); denser than water; used in complex RDs.

**Legend**:

• **Viscosity (cSt)**: higher viscosity reduces emulsification but complicates injection/removal

• **Specific gravity**: standard SO (< 1) floats, heavy SO (> 1) sinks

• **Surface tension**: contributes to retinal adhesion; relatively similar across formulations

• **Refractive index**: ~1.404, slightly higher than aqueous, producing hyperopic shift in pseudophakic eyes

Four physical parameters characterize a tamponade agent: specific gravity, buoyancy, interfacial tension, and viscosity [4].

1. Specific gravity, and clinical implications.

Conventional PDMS oils have a specific gravity slightly lower than water (~ 0.97 vs. 1.0), meaning they are less dense and therefore float within the aqueous environment of the vitreous cavity. This property makes them particularly suitable for supporting superior retinal breaks, while their effectiveness is limited in inferior pathologies. In contrast, heavy silicone oils (HSOs) are not chemically bonded compounds but rather physical mixtures of PDMS with denser components, typically semifluorinated alkanes such as perfluorohexyloctane (F6H8). These mixtures achieve a density greater than water (> 1 g/mL), allowing them to sink and provide tamponade for inferior retinal breaks or detachments.

2) Buoyancy and contact pressure:

Although buoyancy is often described using Archimedes’ principle, its role in vitreous tamponade is limited. In a nearly completely oil-filled vitreous cavity, the volume of displaced fluid is minimal, and therefore the net buoyant force is negligible. The retinal tamponade effect is better explained by the direct mechanical apposition (contact) between the tamponade bubble and the retinal surface. This contact pressure depends primarily on the shape and volume of the bubble, the relative density between tamponade and residual intraocular fluid, and gravitational orientation of the eye. Compared with intraocular gases, which have a much lower density, silicone oil generates lower effective contact forces, contributing to its different tamponade profile.

3) Interfacial tension:

Interfacial tension refers to the force at the interface between two immiscible fluids, such as silicone oil and aqueous humor. Silicone oils have lower interfacial tension (~ 40–45 mN/m) compared with intraocular gases (~ 80 mN/m). This property has direct surgical implications: (1) lower interfacial tension facilitates deformation of the oil bubble, influencing the shape of the fluid–oil meniscus at retinal breaks; (2) it increases the risk of leakage through sclerotomies, often necessitating careful wound construction or suturing; (3) it may contribute to instability of the tamponade interface, particularly in complex retinal detachments.

4) Viscosity:

Viscosity reflects the resistance of a fluid to deformation under shear stress. Silicone oils are available in a range of viscosities (typically 1000–5000 cSt), which are directly determined by PDMS chain length and molecular weight. From a clinical perspective: lower-viscosity oils (~ 1000 cSt) are easier to inject and remove but show a higher tendency to emulsify; higher-viscosity oils (~ 5000 cSt or higher) are more resistant to emulsification but require greater surgical effort for injection and removal [5].

### Composition and surgical considerations of heavy silicon oil

Chemical characteristics directly determine the physical behavior of silicone oils and, consequently, their clinical applications and limitations. Regarding viscosity, low-viscosity oils (approximately 1,000 cSt) are easier to inject and remove but exhibit a greater tendency to emulsify. High-viscosity oils (generally 5,000 cSt or higher) provide better resistance to emulsification but require greater surgical effort during both injection and extraction. HSOs may adhere to the retinal surface, particularly in areas of retinal damage, inflammation, or proliferative vitreoretinopathy, making complete aspiration challenging and increasing the risk of iatrogenic retinal trauma. In addition, emulsified droplets may become trapped in retinal folds or subretinal spaces, further complicating removal. Previous studies have reported a higher incidence of incomplete removal and retinal adherence with heavy silicone oils compared with conventional PDMS [6]. Slow active aspiration, repeated fluid–air exchanges, and gentle retinal brushing may reduce residual oil and minimize retinal trauma; moreover, careful postoperative monitoring is essential for early detection and management of associated complications [7]. Specific gravity distinguishes different uses of SOs. Standard SOs (polydimethylsiloxane, PDMS) are lighter than water, making them effective for treating superior retinal breaks but less ideal for inferior pathologies, whereas heavy silicone oils (HSOs) are composed of PDMS combined with denser agents, typically semifluorinated alkanes such as perfluorohexyloctane (F6H8).” With a density greater than 1 g/mL, these agents are suitable for tamponing inferior retinal breaks or detachments, as in products such as Densiron 68→ and Oxane HDⓇ. Regarding chemical composition, pure PDMS represents the traditional silicone oil used as a standard tamponade; blends or copolymers, including mixtures with semifluorinated alkanes (F6H8) or other additives, are designed to enhance the density or alter the physical properties. Modified SOs are experimental or recently developed formulations intended to improve biocompatibility, reduce emulsification, or achieve specific rheological characteristics (Fig. 2).

### Biocompatibility

A critical factor in tamponade selection is its potential to induce an immune-mediated inflammatory response in the host. This is particularly true for SOs, which are implantable devices and long-term surgically invasive devices, as their contact with the eye exceeds 30 days (class IIb), according to EU regulations [8, 9]. The CE marking (Conformité Européenne) certifies compliance with EU standards for safety, efficacy, and regulation, requiring manufacturers to undergo conformity assessment and periodic audits [8]. Moreover, ISO standards, such as ISO 13485:2016 (quality management system) and ISO 16672:2020 (ocular tamponades), define the safety, performance, and manufacturing requirements for SOs [9, 10].

### Toxicity

SOs are industrial products obtained through chemical synthesis. As previously described, SOs are marketed according to their viscosity (e.g., 1000 cSt or 5000 cSt), which reflects the predominant polymeric fraction composed of chains of the intended length [11]. However, the synthesis process inevitably yields silicone chains with both higher and lower degrees of polymerization. Despite the subsequent purification steps, the final product is then a mixture of polymers and oligomers with varying chain lengths.

SOs contain impurities such as PDMS chains with molecular weights (M) ≤ 10,000 g/mol, including both linear and cyclic low-molecular-weight components (LMWC) with M ≤ 1000 g/mol, as well as residual catalysts [12]. These impurities originate from the PDMS synthesis process, which generates siloxanes of heterogeneous chain lengths and molecular weights.

Even with purification and ultrapurification, these low-molecular-weight fractions are not eliminated, and some of them are toxic, being capable of diffusing into ocular tissues [13]. SO emulsification can elicit a macrophage-mediated foreign body reaction, potentially leading to retinal inflammation and necrosis [14]. This process is further amplified by the presence of LMWC and biosurfactants, including plasma lipoproteins, high-density lipoprotein (HDL) apolipoproteins, and red blood cell membranes [15, 16]. Romano et al. investigated the potential toxicity of SO in BALB 3T3 fibroblasts and human RPE cells (ARPE-19). This study assessed three different forms of conventional SOs (1000 cSt): raw, intermediate (obtained during distillation), and purified (final product after distillation), along with a concentrated LMWC. The results demonstrated no evidence of acute toxicity at 24 h. However, prolonged exposure to less purified SO and LMWC concentrate may contribute to reduced cell viability, suggesting potential concerns for chronic toxicity [17].

### Mechanistic insights into silicone oil emulsification

**Fig. 3:**
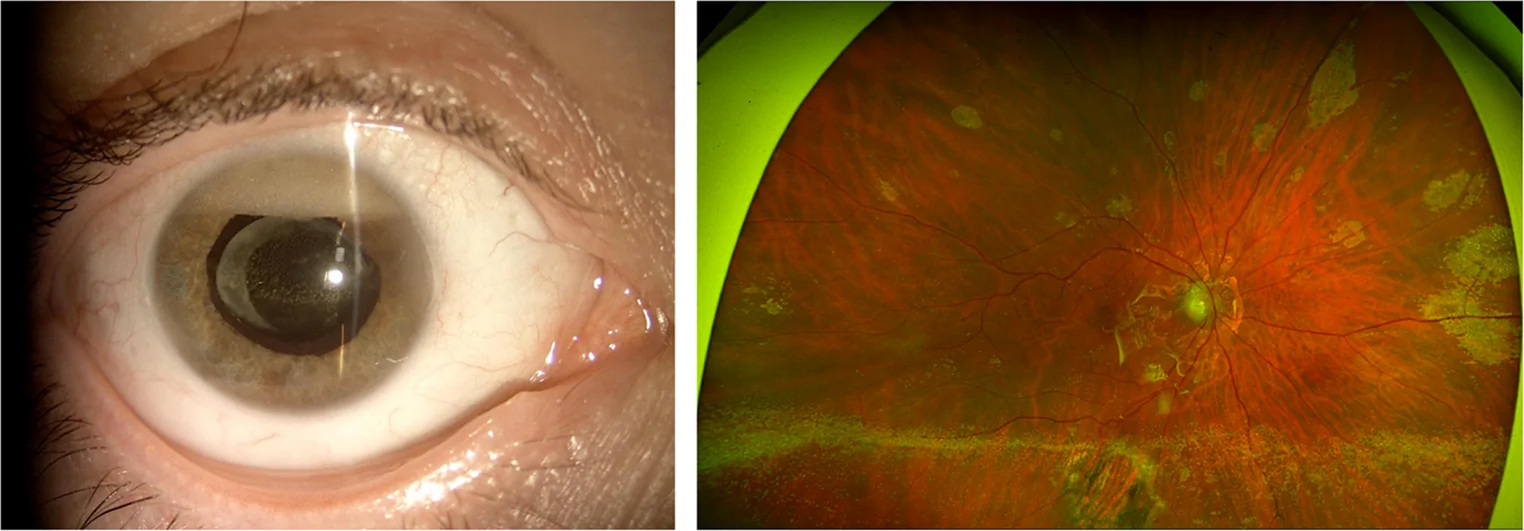
Representative clinical examples of intraocular silicone oil emulsification

Another major determinant of SO-related morbidity is its physical emulsification within the ocular environment. Emulsification is a physical process in which microdroplets bud off from the main SO bubble. Clinically significant emulsification most commonly occurs in eyes requiring prolonged tamponade, particularly in the presence of extensive retinal pathology, postoperative inflammation, or repeated intraocular surgical manipulation. Diagnosis is primarily clinical, relying on slit-lamp examination to detect the presence of SO micro-droplets. Efforts have been made to enhance in vivo detection using OCT, ultra-widefield fundus imaging, and MRI [18], however, this phenomenon extends far beyond what can be visualized clinically (Fig. 3). Analyses of aqueous samples collected at the time of SO removal, employing techniques such as Coulter counting and laser light scattering, have consistently revealed emulsified droplets, many smaller than 2 μm, well below the detection threshold of gonioscopy or slit-lamp biomicroscopy [19]. Several risk factors have been associated with increased SO emulsification, including longer tamponade duration, lower viscosity oils, postoperative inflammation, intraocular hemorrhage, residual vitreous scaffold, frequent eye movements, aphakia or large posterior capsulotomies, and elevated intraocular pressure. Mechanical shear forces at the oil–aqueous interface and the presence of endogenous surfactants such as serum proteins further promote droplet formation [20]. Certain complications related to SO emulsification cannot be adequately resolved through standard SO removal [21], or by performing triple fluid–air exchanges intended to enhance droplet clearance [22]. In fact, the use of air may paradoxically promote adherence of emulsified droplets to the retinal surface [23]. Perfluorobutylpentane (F4H5) has been investigated as an intraoperative solvent for residual SO because of its excellent miscibility with oil [24]. However, due to its hydrophobic nature, F4H5 is ineffective in dissolving emulsified droplets suspended within the aqueous phase. To overcome this limitation, a more efficient rinsing strategy has been proposed, combining a volatile liquid (such as LMW-SO or F4H5) with a hydrophobic surfactant [25] (Table 2).

**Table 2:** Summary of emerging solvent-assisted approaches for intraoperative removal of silicone oil (SO) droplets. Techniques differ in agent, miscibility, and mechanism, with early evidence suggesting improved clearance compared to saline. While some anterior-chamber applications show short-term safety, most strategies remain supported only by preclinical or limited clinical data, and in vivo validation and regulatory approval are still lacking

Approach	Agent(s)	Mechanism	Evidence level	Advantages	Limitations / Safety
**Anterior-chamber rinse**	F4H5 (perfluorobutylpentane)	Solubilizes SO droplets, flushes angle/AC	Pilot clinical study (2023, GDD cases)	Significant ↓ IOP, reduced AC droplets, endothelial safety at 12 mo	Only AC tested; limited data; off-label
**Posterior-segment rinse**	F4H5	Miscibility with SO allows “solvent-assisted” removal under fluid	Preclinical + early clinical reports	Simple protocol, miscible with SO	Minimal in vivo data; avoid prolonged macular contact
**Double-emulsification rinse**	F4H5 + PFPE-PEG-PFPE surfactant	Encapsulation of SO droplets into O/W/O double emulsion → aspiration	Ex vivo + in vitro (Chan 2018)	Markedly ↓ residue vs. saline; in vitro biocompatibility	Not yet tested in vivo; regulatory hurdles
**Alternative SFA**	F6H8 (perfluorohexyloctane)	Miscible with SO; historically used intraop and in HSO	Case reports / historical	Known handling properties; dissolves sticky SO on IOL	Reports of inflammation; less effective than F4H5 for droplet washout
**LMW-SO cleaners**	Volatile low-MW oils + surfactant	Volatile solvent encapsulates droplets (double emulsification)	ARVO abstracts, lab data	Greater clearance than saline in 3D/ex vivo	No in vivo data; theoretical ocular toxicity unknown

### SOs and intravitreal injections

As intravitreal therapy has become essential to retinal disease management, interactions between SO and concurrently administered intraocular drugs have become increasingly relevant, raising important pharmacological considerations. The main challenge lies in understanding drug distribution, metabolism, and clearance, given that SO is a lipophilic medium, whereas the native vitreous is predominantly hydrophilic [26].

Preclinical research, case studies, and small series suggest that anti-VEGF agents have pharmacokinetics comparable to those in a normal vitreous environment, supporting the use of standard doses of bevacizumab (1.25 mg), aflibercept (2 mg), and ranibizumab (0.5 mg) in SO-filled eyes. Among corticosteroids, dexamethasone implants (0.7 mg) appear both effective and safe, although data on fluocinolone acetonide (0.19 mg) remain limited. Intravitreal methotrexate (250–400 µg) has shown some therapeutic effect, though keratopathy has been reported as a potential adverse event. Antiviral drugs such as foscarnet (1.2–2.4 mg/0.1 ml) and ganciclovir (4 mg/0.1 ml), as well as antibiotics like piperacillin/tazobactam (250 µg/0.1 ml), have been administered with acceptable tolerability. Current guidelines recommend maintaining standard dosing regimens in eyes filled with SO, while emphasizing the need for vigilant clinical monitoring and larger prospective studies to improve the evidence base [27].

### Complications of SO use

Although SO is an effective tamponade agent, its use is associated with a spectrum of ocular and systemic complications.

### Ocular complications

#### Surgery-related complications

One of the most common technical challenges is achieving an optimal SO fill. Because SO tends to form a large spherical bubble, a thin fluid layer may persist between the oil and the retinal surface. For maximal tamponade effect, the vitreous cavity should be nearly completely filled [28]. Underfilling can leave unsupported retinal areas, predisposing to recurrent RD and PVR [29]. Conversely, overfilling may induce anterior rotation of the iridociliary diaphragm, resulting in elevated IOP, shallowing of the anterior chamber, or even iris prolapse through the corneal wound. These issues can often be avoided through careful intraoperative monitoring—for example, by observing the vascular pulse within the optic disc during SO injection until a small air bubble appears, followed by completion of the fill while allowing passive drainage of residual air [30].

SO may also migrate from the vitreous cavity into adjacent ocular or periocular structures. It can escape through sclerotomies, perforation sites, or glaucoma drainage devices into the subconjunctival space or enter the subretinal space in eyes with residual, contracted retinas. Preventive measures include meticulous wound closure, thorough membrane peeling, and, when necessary, adjunctive techniques such as scleral buckling or retinectomy [31, 32]. Another recognized issue is the adherence of SO microdroplets to intraocular lenses (IOLs), which can impair vision, particularly after SOR. In some cases, IOL exchange may be required [33]. Material choice can influence this risk—acrylic and polymethyl methacrylate IOLs appear less prone to SO droplet adherence than other lens types [34].

#### Postoperative complications

Postoperative complications associated with SO tamponade are multifactorial and largely related to its long-term intraocular persistence, rather than to a single isolated mechanism. In the long term, complications associated with SO are largely attributable to emulsification, which can compromise ocular structures. Emulsification is a process in which the cohesive oil mass breaks down into dispersed microdroplets over time, driven primarily by reduced surface tension facilitated by the presence of surfactants such as endogenous proteins, phospholipids, and lipoproteins. Higher-viscosity oils (e.g.; 5000 cSt) exhibit a lower tendency to emulsify than lower-viscosity oils (e.g.;1000 cSt or HSO) [35]. Other factors influencing the risk of emulsification include the duration of tamponade, aphakia, high myopia, insufficient filling of the vitreous cavity, and excessive ocular movements, as observed in patients with nystagmus [35, 36]. Lu et al. recently suggested that emulsification may also be triggered by the breakup of PDMS chains adhering to the retinal surface and subsequently disrupted by ocular movements. Although such PDMS–retina adhesive forces are rarely encountered under normal in vivo conditions, they may become significant when perfluorocarbon liquids are used adjunctively [37, 38].

Corneal decompensation is among the most frequent complications, typically arising when the SO remains in prolonged contact with the corneal endothelium. Over time, this contact causes endothelial cell loss, leading to corneal edema and, in advanced stages, bullous keratopathy [3]. The risk is particularly high in eyes where oil migrates into the anterior chamber, a scenario that occurs more readily in aphakic patients or when emulsification develops. Eyes with pre-existing corneal pathologies are particularly vulnerable. To mitigate this, surgeons aim to limit tamponade duration, remove the oil promptly when indicated, and adopt intraoperative measures such as performing a peripheral iridectomy in aphakic eyes to prevent pupillary block and anterior migration of the oil [30].

Alterations in IOP represent another significant concern. Postoperative IOP elevation, reported in up to 40% of patients, may result from several mechanisms, including SO overfilling, pupillary block, inflammatory responses, emulsification, anterior chamber migration of the oil, or exacerbation of pre-existing glaucoma [39, 40]. Although initial pressure spikes are often managed medically, persistent elevation can lead to secondary glaucoma, sometimes requiring surgical intervention. In refractory cases, glaucoma drainage devices or oil removal may be necessary; placing drainage implants in the inferior anterior chamber can help avoid tube blockage by oil [41]. Conversely, hypotony, although less common, can occur due to ciliary body detachment, chronic RD, or wound leakage at sclerotomy sites, and may require wound revision or reinjection of SO to restore adequate IOP [42].

A particularly feared complication is optic neuropathy, which, though rare, can have devastating visual consequences. Prolonged tamponade, sustained IOP elevation, SO migration into the optic nerve, ischemia, and potential oil-related toxicity have all been implicated. Careful control of IOP during infusion and close postoperative monitoring in eyes with pre-existing optic nerve pathology are therefore essential [43].

An increasingly recognized complication in recent years is unexplained visual loss (UVL) following SO tamponade with reported prevalence ranging from approximately 3% to 30%, depending on case definition, duration of tamponade and study design. It typically presents within the first six weeks after surgery as a central scotoma with a decrease of two or more Snellen lines without an identifiable structural cause. Preoperative characteristics associated with UVL include macula-on retinal detachment, relatively preserved baseline visual acuity, younger age, prolonged SO tamponade, and higher intraocular pressure during follow-up. Some studies have also suggested that fovea-sparing detachments and primary vitrectomy with SO may predispose to this condition, possibly because a structurally intact macula is more vulnerable to toxic or mechanical insults [44]. Hypotheses for its pathogenesis include direct damage from oil microdroplets penetrating the retina, phototoxicity related to SO transparency, or inflammatory injury mediated by cytokines trapped between the oil and the retinal surface [45]. Structural findings on OCT are variable and may reveal thinning of the ganglion cell complex or microcystic changes, while functional testing highlights central scotomas on microperimetry or perimetry. Electrophysiological studies suggest macular and optic nerve dysfunction but lack disease-specific signatures. The long-term prognosis is generally poor, although occasional partial recoveries have been documented. Given the absence of preventive or therapeutic strategies, awareness of this condition is crucial for surgical planning and patient counseling. Further studies focusing on intraoperative dynamics, SO composition, and postoperative functional recovery are needed to elucidate underlying mechanisms and guide prevention [46]. Further investigations into SO–retina interactions and postoperative retinal microstructure are warranted [47].

Cataract progression is the most common cause of postoperative visual acuity decline after PPV. The risk is particularly high for nuclear sclerosis, which tends to develop or accelerate following surgery due to the increased intraocular oxygen tension within the vitreous cavity. Several studies have reported that up to 80% of phakic eyes develop clinically significant cataracts within two years of vitrectomy. Given this high likelihood, many vitreoretinal surgeons advocate combined phacovitrectomy during the primary intervention, particularly in patients over 50 years of age, to avoid the need for subsequent cataract surgery. This strategy reduces the total number of procedures, provides an immediate, clear postoperative fundus view, and facilitates long-term retinal monitoring [48].

OCT assessment of macular alterations in silicone oil–filled eyes primarily provides prognostic information, allowing correlation between the integrity of the outer retinal layers (EZ, ELM, ONL) and postoperative visual acuity. As reported by Saber et al., the degree of disruption of these layers is strongly associated with visual recovery. However, in this context, silicone oil represents the tamponade used in more complex cases rather than an independent variable directly responsible for the observed damage. Therefore, the structural alterations mainly reflect the extent of photoreceptor injury related to macula-off retinal detachment and surgical repair. Nevertheless, a potential contributory role of silicone oil—particularly in relation to tamponade duration, inflammation, or emulsification—cannot be completely excluded [49]. When cystoid macular edema is present, it can be managed with sub-Tenon corticosteroid injections, intravitreal dexamethasone implants, or anti-VEGF agents. Collectively, these postoperative complications highlight the importance of careful patient selection, meticulous surgical technique, and close follow-up to optimize both anatomical and functional outcomes after SO use [50].

#### Systemic complications

Although SOs are primarily confined to the intraocular cavity, their extravasation beyond ocular boundaries can have deleterious systemic effects. Silicone Embolism Syndrome (SES) is a rare systemic complication associated with intraocular SO use. SES results from the extravasation of SO into the systemic circulation, potentially leading to life-threatening consequences, such as pulmonary embolism, neurological deficits, and multiorgan dysfunction [51].

The exact pathogenesis of this syndrome is not fully understood; however, several mechanisms have been proposed. Experimental and histopathological studies have shown SO droplets within the optic nerve head and subarachnoid space, extending into lateral ventricles system, suggesting retrograde migration along the optic nerve sheath as a potential route of systemic entry. Disruption of the blood–retinal and blood–optic nerve barriers, particularly in the presence of elevated intraocular pressure, optic disc anomalies, or postoperative inflammation, may facilitate translocation of emulsified SO droplets into the cerebrospinal fluid and subsequently into the venous circulation. Low-viscosity SOs exhibit a higher tendency to migrate owing to their reduced mechanical resistance, which may facilitate passage across physiological barriers [52]. Anecdotal reports also describe cases of SES associated with high viscosity SO tamponade, in which uncontrolled IOP and optic disc anomalies were implicated, emphasizing the importance of timely oil removal and careful postoperative monitoring [53].

### Surgical indication for the use of SO

**Fig. 4:**
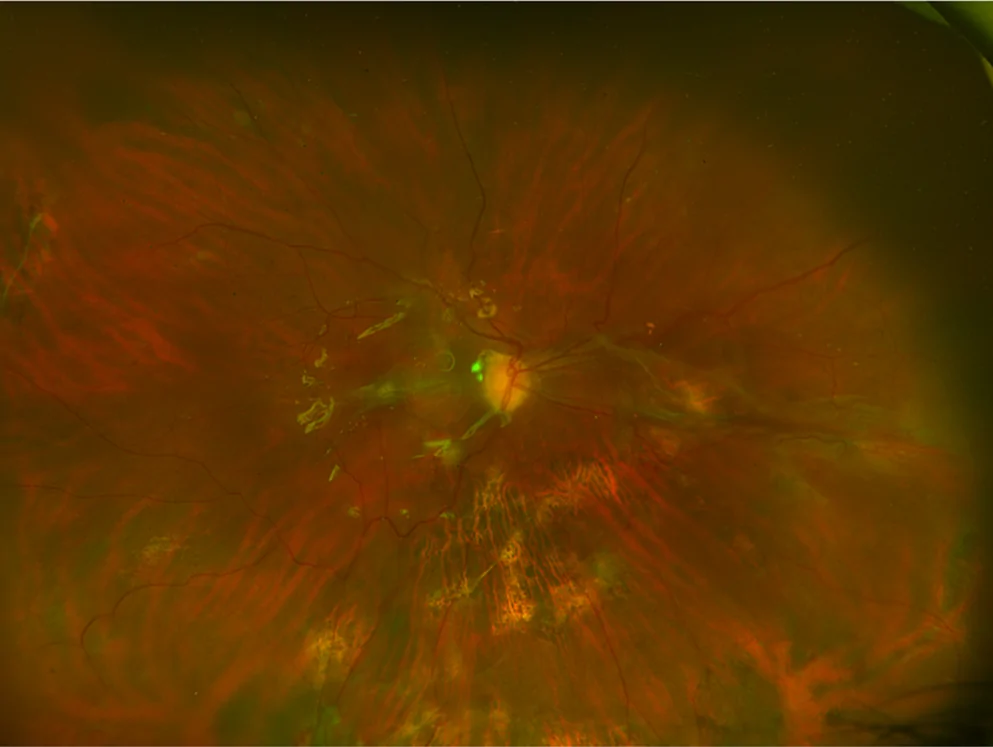
Ultra-widefield fundus photograph of an eye with polydimethylsiloxane (PDMS) tamponade following surgery for rhegmatogenous retinal detachment complicated by proliferative vitreoretinopathy (PVR). The image shows a silicone oil–filled vitreous cavity with residual retinal folds and fibrotic membranes consistent with advanced PVR

SO is commonly employed as a long-term intraocular tamponade in complex RD surgeries, particularly in cases at high risk of PVR, giant retinal tears, or recurrent RD following a previous surgical failure. SO is also frequently indicated in traumatic RD, including those complicated by intraocular foreign bodies or concomitant open-globe injuries, as it provides mechanical stabilization while allowing for subsequent surgical procedures if required (Fig. 4).

SO is additionally preferred in patients for whom postoperative positioning is not feasible, such as individuals with physical or cognitive limitations, those residing at high altitudes, or those requiring early air travel, situations in which intraocular gas expansion may pose a risk. It is also advantageous in cases involving inferior retinal breaks, where the buoyancy of gas tamponades may be insufficient to ensure adequate retinal support.

Several multicenter studies have evaluated the use of HSO as an intraocular tamponade for managing inferior RDs associated with PVR. In a recent cohort of 139 eyes with PVR-related RD, 7.2% presented with primary RD and inferior PVR, whereas the majority (92.8%) had recurrent RD with inferior PVR. Notably, 73.9% of these eyes had previously received conventional SO tamponade before HSO use. At the time of HSO removal, retinal reattachment was achieved in 87.6% of cases, while 12.4% of patients showed persistent detachment despite tamponade. During follow-up, 23.2% of eyes experienced recurrent RD. Importantly, the rate of postoperative re-detachment was significantly influenced by the retinal status at the time of HSO removal: only 14.2% of eyes with an attached retina at removal developed re-detachment, compared with 88.2% of those with persistent RD. Conversely, early HSO removal followed by short-acting tamponades (e.g., air or gas), rather than silicone oil, together with shorter HSO tamponade duration, was associated with an increased risk of recurrence. These findings underscore the importance of both adequate HSO retention time and appropriate choice of the subsequent endotamponade, highlighting the need for careful tamponade planning during the postoperative phase [54].

As reported by Romano et al., heavy silicone oil (HSO) may reduce the need for scleral buckling in inferior breaks, as Densiron-68 provides effective inferior tamponade [55]. Boscia et al. compared HSO with silicone oil 1300 cSt plus scleral buckle in rhegmatogenous retinal detachment complicated by PVR and inferior retinal breaks, finding no significant differences in anatomical or functional outcomes; however, the combined oil–buckle approach increases surgical time, despite the buckle offering posterior displacement of the ora serrata and improved support for posterior breaks [56]. On the other hand, HSO creates a relative “empty space” in superior quadrants, potentially favoring fluid and cellular accumulation and PVR recurrence, with Wolf et al. reporting superior re-detachment in 18% of severe PVR cases, consistent with our single recurrence due to a superior tear [57]. Finally, the limited epiretinal membrane formation observed in the Oxane HD group may be related to the shorter intraocular retention time, as prolonged HSO tamponade has been associated with higher membrane rates in previous series [58]. Although silicone oil remains essential in complex retinal detachments, especially in the presence of advanced proliferative vitreoretinopathy or inferior pathology, several studies have suggested poorer functional outcomes compared with gas tamponade in selected cases. In a propensity score–matched analysis from the Japan Retinal Detachment Registry, Funatsu et al. reported worse postoperative visual outcomes in eyes treated with silicone oil despite anatomical success, emphasizing the importance of careful tamponade selection and individualized surgical planning [59].

The Endophthalmitis Vitrectomy Study (EVS) demonstrated the superiority of primary pars plana vitrectomy (PPV) over tap-and-inject approaches in managing acute post-cataract endophthalmitis, particularly among patients with only light perception (LP) vision. In this subgroup, 33% of those who underwent PPV achieved a final visual acuity of 20/40 or better, compared with 11% in the tap-and-inject group. In contrast, among patients with an initial visual acuity better than LP, PPV conferred no significant advantage. However, the EVS did not specify the optimal choice of intraocular tamponade following PPV [60]. More recent evidence suggests that SO may be particularly advantageous in selected cases, as it combines antimicrobial activity—limiting intraocular bacterial proliferation—with retinal protection, particularly in the presence of undetected retinal breaks. Furthermore, SO provides a stable and optically clear medium during the early postoperative period [61].

A retrospective study by Yang et al. involving 19 eyes with no light perception (NLP) following severe ocular trauma demonstrated that, owing to advancements in PPV techniques and the use of SO tamponade, a substantial proportion of patients achieved partial visual recovery within one year. These findings indicate that NLP after trauma does not necessarily imply irreversible vision loss. As long as the posterior pole, optic nerve, and visual pathways remain intact, traumatized eyes may retain the potential for meaningful visual improvement. SO provides a stable internal tamponade, prevents recurrent RD, particularly in the presence of undetected retinal breaks or PVR, and mitigates post-operative complications such as recurrent vitreous hemorrhage, persistent retinal traction, and intraocular inflammation. Additionally, SO enables more effective 360-degree prophylactic laser retinopexy prior to oil removal and supports faster visual rehabilitation owing to its optical transparency [62].

SO tamponade is also widely employed in the surgical management of tractional RD secondary to proliferative diabetic retinopathy (PDR), particularly in complex or advanced cases. Parikh et al. reported that vitrectomy with SO tamponade achieved stable anatomic outcomes and preserved visual function in eyes otherwise at high risk of re-detachment, although complications such as cataract progression, preretinal fibrosis, recurrent RD, anterior chamber migration, corneal edema, and emulsification were relatively common [63]. In cases with a high risk of recurrent RD, extended SO tamponade yielded favorable long-term reattachment rates, even though visual recovery was constrained by preoperative ischemia, macular involvement, and baseline visual acuity [64, 65].

Recurrent retinal detachment after SOR should be considered a surgical outcome rather than a complication directly attributable to silicone oil itself. Reported rates range between 8% and 12% [66]. In a meta-analysis of 16 studies, He et al. identified predictors of RD after SOR. Aphakia (OR, 1.50), high myopia (OR, 2.47), prior failed RD surgery (OR, 1.71), and ocular trauma (OR, 3.52) were associated with an increased risk, whereas 360° peripheral laser retinopexy (OR, 0.40) and encircling scleral band/buckle (OR, 0.58) were protective. The authors conclude that these patient- and eye-specific factors should guide counseling and postoperative surveillance after SOR, and that selective use of 360° laser and/or an encircling band may help reduce the likelihood of retinal re-detachment [67].

### Optimizing the choice of silicone oil in vitreoretinal surgery

Although specific clinical evidence remains limited, several comparative studies have evaluated the performance of different SOs in relation to their physical characteristics, particularly viscosity and specific gravity.

In this context, Soheilian et al. conducted a retrospective study evaluating postoperative outcomes after removal of 1000 and 5000 cSt silicone oil in 82 eyes with complex retinal detachment and reported that poorer baseline visual acuity (≤ 6/120) and tamponade with 5000 cSt oil were associated with a significantly higher risk of final visual acuity ≤ 6/120, while retinal re-detachment occurred more frequently in the 5000 cSt group, particularly in the presence of proliferative vitreoretinopathy. However, it should be noted that the 5000 cSt group included a higher proportion of more complex cases (85% with IOFB or PVR vs. 70% in the 1000 cSt group) and was associated with a longer tamponade duration, which may have influenced the observed outcomes [68]. In contrast, Scott et al. found comparable anatomical and functional outcomes between 1000 and 5000 cSt silicone oil. Taken together, these conflicting results suggest that the impact of silicone oil viscosity on postoperative outcomes remains controversial and may be influenced by baseline disease severity and surgical context [69].

Moussa et al. performed a retrospective cohort study of 259 eyes with primary rhegmatogenous RD (RRD), comparing standard SO with HSO (Densiron). No significant differences were found in anatomical success, visual improvement, PVR incidence, or the need for glaucoma surgery between the tamponade types. However, the use of standard SO was independently associated with a significantly higher likelihood of requiring retinectomy (OR, 15.3; *p* = 0.011). Tamponade duration had no measurable impact on any of the assessed outcomes. These data indicate that Densiron provides comparable efficacy and safety, with a potential advantage in reducing the need for retinectomy in primary RRD surgery [70].

Hammer et al. reported a retrospective series of 20 eyes undergoing vitrectomy for recurrent inferior PVR using HSO (Densiron 68→). Although the use of heavy tamponades has largely been abandoned in primary RD because of their unfavorable complication profile, they still retain value in the management of recurrent cases with inferior PVR [71]. Complications were classified as related to IOP, inflammation, lens status, and oil emulsification or migration. Elevated IOP was mainly observed within the first 15 postoperative days, but its incidence was comparable to that reported with conventional silicone oil. Regarding inflammation, prior laboratory studies suggested that HSO-containing semifluorinated alkanes may be more pro-inflammatory, as their greater lipophilicity facilitates retinal penetration. However, Hammer et al. did not observe an higher rate of inflammatory complications with Densiron 68Ⓡ, hypothesizing that the average tamponade duration of four months might have been too short to reveal such pro-inflammatory effects [72]. Consistently, Semeraro et al. demonstrated that prostaglandin-E2 and interleukin-1- α levels increased significantly with longer HSO tamponade duration [13]. Thus, the use of HSO in cases requiring inferior tamponade appears to offer a safety profile comparable to that of conventional lighter oils [13, 72].

### Timing of silicone oil removal

Kampik et al. proposed strategies and indications for SOR. Factors influencing the decision to maintain or remove SO include the persistence of the underlying disease process with the associated risk of retinal re-detachment, the stability and quiescence of the retina, maintenance of visual acuity, and the emergence of secondary complications such as macular pucker or elevated IOP. Additional considerations include the development of keratopathy, particularly in cases where SO migrates into the anterior chamber, the formation of secondary cataracts, and the progressive SO emulsification over time [73].

Several studies have reported significant changes in central macular thickness following silicone oil removal, ranging from transient retinal thickening to persistent central macular thinning, both of which may negatively affect visual recovery. These alterations are likely multifactorial, involving inflammatory rebound, Müller cell dysfunction, retinal toxicity, and mechanical stress, underscoring the importance of individualized timing of oil removal and careful postoperative OCT surveillance.

Dubroux et al. performed a retrospective analysis to assess the effect of SO tamponade duration on retinal structure following surgery for RRD. The study included 68 patients, evenly divided into two groups according to whether SO was removed before or after six months postoperatively. In these eyes, the authors measured central macular thickness (CMT), inner retinal layer thickness (IRLT), and outer retinal layer thickness (ORLT). They observed that while the oil remained in place, both CMT and IRLT became significantly thinner, whereas ORLT showed no relevant modifications. After SO removal, both groups exhibited a significant increase in CMT (*p* = 0.009 for Group 1; *p* = 0.007 for Group 2) and IRLT (*p* = 0.014 and *p* = 0.013, respectively), whereas ORLT did not change significantly (*p* = 0.080 and *p* = 0.257). The authors concluded that the timing of SOR did not significantly affect anatomical or functional outcomes. These results suggest a certain degree of flexibility in determining tamponade duration, emphasizing the need to tailor decisions to the specific clinical conditions of each patient [74].

The optimal timing of removal is individualized based on anatomical stability, underlying pathologies, and risks of complications. Common complications associated with SO use include corneal decompensation, glaucoma, cataract progression, and recurrent RD. Rare but serious complications, such as silicone emulsification syndrome, involve the migration of SO into systemic circulation.

Abu-Yaghi et al. investigated the outcomes of extended SO tamponade in RD surgery. This study highlighted that prolonged use of SO can lead to complications such as ocular hypertension, keratopathy secondary to oil emulsification, recurrent RD, and epiretinal membrane formation. These findings underscore the importance of the timely SOR to mitigate potential adverse effects. The study included 85 eyes of 85 patients who underwent PPV with a SO tamponade and were divided into two groups based on the viscosity of the silicone oil used (1000 and 5000 cSt). The results demonstrated that both groups experienced similar long-term complications, reinforcing the need for careful management of tamponade duration. In conclusion, the authors suggested that SO should ideally be removed within 3–6 months to prevent or minimize these complications [30].

In eyes that have already undergone multiple vitreoretinal procedures, SO can be used as a permanent or extended tamponade. For instance, in particularly challenging cases of RD, SO has proven effective in maintaining long-term retinal reattachment and may therefore represent a viable therapeutic option when other approaches are insufficient. However, the prolonged intraocular presence of SO is not without risk. Complications such as emulsification, secondary glaucoma, corneal decompensation, cataract formation, and other oil-related changes are relatively common, and their incidence increases with tamponade duration. Vidne-Hay et al. reported that despite these adverse events, long-term tamponade may still provide functional benefits and preserve useful vision in selected eyes with advanced proliferative vitreoretinopathy, recurrent retinal detachment after multiple prior surgeries, extensive inferior retinal pathology or large retinectomies, hypotony-prone eyes, and in patients unable to maintain postoperative positioning. Nevertheless, this approach requires careful patient selection and close monitoring, weighing the advantage of sustained retinal attachment against the progressive risk of oil-related complications [75].

### Future perspectives

#### Emerging concerns and future restrictions of SO use

The European Union recently included perfluorohexanoic acid (PFHxA) and related compounds in Annex XVII of REACH (Regulation [EU] 2024/2462), introducing new restrictions on their use. This regulation will prohibit the use of PFHxA in multiple consumer product categories as of October 10, 2026. Notably, medical devices regulated under the MDR and IVDR are explicitly exempt, meaning that intraocular tamponades are not directly affected by this restriction.

On August 20, 2025, the EU published an updated proposal for a “universal” PFAS restriction under REACH, ranging from an almost complete ban to time-limited derogations.

Within this framework, standard SOs (PDMS 1,000–5,000 cSt) are unlikely to be affected, as they lack C–F bonds and therefore fall outside the current PFAS definitions. In contrast, HSOs such as Densiron 68 (PDMS + perfluorohexyloctane/F6H8) and Oxane HD/5700 (PDMS + RMN3, a partially fluorinated olefin) are within the scope because they contain semifluorinated or partially fluorinated moieties.

Perfluorocarbon liquids (PFCLs) used intraoperatively (e.g., perfluorodecalin) are more likely to fall within the scope of broader PFAS restrictions due to their perfluorinated chemistry. For these agents, essential-use exemptions may be introduced in the future to ensure clinical availability where no suitable alternatives exist.

#### New frontiers in the development of tamponades

The “ideal” SO would balance handling and stability characteristics: it should have low apparent viscosity under surgical shear to facilitate injection and aspiration through small-gauge cannulas during surgery, while maintaining high zero-shear viscosity and interfacial stability when at rest to prevent droplet formation and persistence [76].

The foldable capsular vitreous body (FCVB) is an artificial vitreous substitute composed of a transparent, flexible silicone capsule implanted into the vitreous cavity through a 4-mm pars plana incision after vitrectomy. After placement, the capsule is inflated with a balanced solution through a self-sealing valve positioned beneath the conjunctiva, providing long-term retinal tamponade and mechanical support. By isolating the filling agent from direct contact with intraocular tissues, the FCVB aims to reduce complications commonly seen with conventional tamponades, including SO emulsification and toxicity. In a systematic review, Serhan et al. analyzed 19 studies encompassing more than 1000 implanted eyes. The most frequent indications were severe ocular trauma (approximately 60–70% of cases), followed by recurrent or complex retinal detachment (20–25%) and SO–dependent eyes (approximately 10%) [76]. The reported mean follow-up periods ranged from 6 to 36 months. Overall, FCVB implantation demonstrated a high rate of globe preservation, with anatomical integrity maintained in more than 80% of cases. However, visual outcomes were modest; in most series, fewer than 30% of patients achieved a gain of two or more lines of visual acuity, with functional recovery largely limited by the severity of the underlying retinal damage. Adverse events were relatively frequent, though most were mild and manageable. Elevated intraocular pressure was observed in 15–25% of cases, corneal complications such as edema or decompensation in 10–15% of cases, and inflammatory reactions in 8–12% of cases. Mechanical complications, including capsular opacity or minor deformation, were reported in fewer than 5% of eyes. Notably, emulsification and intraocular migration were virtually absent in contrast to SO. However, functional recovery remains limited, and most published studies are small case series or single-center studies with limited follow-up duration. Larger prospective multicenter trials are required to confirm efficacy, optimize patient selection, and evaluate long-term safety.

### Hydrogels

In RD surgery, early substitutes of the vitreous body were based on non-crosslinked polymer solutions, such as hyaluronic acid (HA) and collagen [77]. However, their intravitreal residence time was very short—approximately 2.1 days for HA, 5.7 days for collagen, and 8.4 days for HA-collagen mixtures. Consequently, these solutions were regarded only as short-term tamponades, since the absence of crosslinking precluded the establishment of stable mechanical homeostasis within the eye [78].

The development of preformed hydrogels, constituted of crosslinked polymers derived from biopolymers (e.g., HA, collagen, and alginate) or synthetic polymers (e.g., PEG, PVA, PVP, and acrylates) provided a longer-term solution. These are characterized by high transparency, appropriate refractive indices, and viscoelastic properties. Moreover, preformed hydrogels offer the possibility of purifying the matrices before injection, thereby reducing the risk of toxicity from residual monomers or crosslinkers [79]. Nevertheless, many preformed hydrogels may undergo structural alteration through the injection through small-bore needles (≤ 25G), resulting in a loss of optical clarity and increased forward light scattering [80]. An alternative strategy involves the use of in situ crosslinked hydrogels, which are injected as liquid precursors. Once in the vitreous cavity, they undergo gelation. These systems can form via chemical crosslinking, posing potential toxicity risks from residual monomers, initiators, or catalysts, or via physical mechanisms triggered by temperature, pH, or ionic changes. Among them, thermoresponsive hydrogels are particularly attractive because they undergo sol-to-gel transition at body temperature without requiring exogenous crosslinkers. However, their clinical potential is restricted by limitations such as excessive stiffness and rapid degradation. In situ crosslinked systems can be further categorized into chemically crosslinked gels (with more durable but potentially cytotoxic bonds) and physically crosslinked gels (biocompatible and shear-thinning, but often less stable over time). Overall, hydrophilic hydrogel-based vitreous substitutes more closely replicate the biophysical properties of the native vitreous than conventional tamponades, such as SOs or gases.

They reproduce key features, including high water content, transparency, and distributed viscoelastic properties, while also offering novel therapeutic opportunities, such as sustained drug delivery (e.g., anti-VEGF, corticosteroids, antiproliferative agents) and even the stimulation of vitreous regeneration [81]. Some HA-based hydrogel candidates have already progressed to early clinical trials. However, several challenges remain to be addressed before widespread clinical adoption can be achieved. These include ensuring long-term stability, preventing intraocular pressure elevation due to swelling pressure, and achieving reproducible large-scale manufacturing. Only a handful of HA-crosslinked systems have demonstrated preliminary human data, and further validation in larger patient cohorts is essential. The main limitations currently hindering the clinical translation of hydrogel vitreous substitutes include:

- The elastic modulus (G′) of formulations remains excessively high. While native vitreous exhibits a G′ of approximately 10 Pa, most candidate hydrogels exceed 100–1000 Pa, raising concerns about mechanical damage to ocular tissues.
- Although the optical transparency (> 95%) and the refractive index (1.336–1.338) of vitreous substitutes are readily achieved, degradation rates are often either too rapid, compromising tamponade function, or too slow, impeding clearance and affecting long-term safety.
- The swelling pressure is a major drawback, as excessive expansion of the hydrogel can precipitate harmful increases in intraocular pressure.
- The need for rigorous purification and stabilization during storage (to avoid pH shifts, oxidation, or loss of functional groups during sterilization) is still a significant limitation.

In conclusion, hydrogel-based vitreous substitutes represent the most promising strategy for replacing current tamponades, including gases and SOs. Nevertheless, their successful clinical translation will depend on further optimization of hydrogel formulations, the establishment of robust long-term safety and efficacy data, and the resolution of key manufacturing and regulatory challenges. If these barriers can be overcome, hydrogels may provide not only a functional vitreous replacement but also novel therapeutic applications with the potential to transform the management of vitreoretinal disease [82] (Table 3).

**Table 3:** Summary of polymer-based vitreous substitute classes, highlighting key material properties, potential advantages, and known safety limitations. Most formulations show promising biocompatibility and tamponade performance in preclinical models, but long-term clinical evidence remains limited

Class / Subclass	Example (Product/Polymer)	Technical Features	Advantages	Limitations
1. Non-crosslinked polymer solutions				
*Hyaluronic acid (HA)*	Hruby 1961; Healon (Denlinger 1980)	t½ ≈ 2.1 d; degradation in 1–2 wks; viscosity MW-dependent	Excellent biocompatibility; well tolerated in primates; transparent	Very short residence; no mechanical stability; high recurrence of RD
*Collagen*	Nakagawa 1997	t½ ≈ 5.7 d	Slightly longer retention vs. HA	Still too short; no structural support
*HA + Collagen mix*	Nakagawa 1997	t½ ≈ 8.4 d	Higher viscosity; better short-term tamponade	Limited stability; no long-term tamponade
*HPMC (2.2%*,* 6 cSt)*	Fernandez-Vigo 1990	Transparent, viscosity decreases rapidly	Easy to handle	Ineffective long-term tamponade
*Methylated collagen*	Zhang 2004	Transparent; RI ≈ water; stable 6 mo in rabbits	Biocompatible, normal ERG	Agglomeration in AC; corneal endothelial damage
*Chitosan (2%)*	Yang 2008	Degrades to glucose + H₂O + CO₂	IOP stable; biodegradable	Mild inflammation in vivo
*PEG (300–400 kDa)*	Pritchard 2011	PEG400 rheologically similar to vitreous; but aggregates; clears in days	Transparent; biocompatible in rabbits	Very short residence; white aggregates impair vision
**2. Preformed hydrogels**				
**2.1. Biopolymers**				
*UVHA (glycidyl methacrylated HA)*	Schramm 2012; Schulz 2020–23	G′ 25–50 Pa; RI 1.3365; >90% transparency; >23 mo degradation in vitro	GMP manufacturing; clinical pilot data (IOP stabilization; preserved retina)	Fragmentation during injection (≤ 25G); limited human data
*Healaflow (BDDE-crosslinked HA)*	Barth 2014–21; Ren 2020	97% water; RI 1.3388; G′ ≈15 Pa; enzymatic degradation 4–5 wks	Effective tamponade in animals and RRD trials (97% reattachment)	Short duration; transient IOP rise; cataract in 12%
*Thiolated HA (disulfide crosslinks)*	Schnichels 2017; Hurst 2022	Transparency 85–98%; G′ 150–663 Pa; stable up to 6 mo	Injectable, softer gels closer to vitreous	Stiffer gels cause retinal stress; short-term only
*Collagen (historic)*	Pruett 1974	Gel fragmentation; strong inflammation	Conceptual pioneer	Severe pain, opacities, poor reattachment
*Methylated collagen (I/III)*	Liang 1998	Transparency > 96%; RI 1.3	Good retinal biocompatibility, normal ERG	Anterior aggregation; endothelial toxicity
*Alginate (CaSO₄ crosslinked)*	Schulz 2020	Transparency > 90%; RI ~ 1.336; G′ 19–74 Pa	Potential long-term replacement (non-degradable in mammals)	Risk of opacity if impurities (endotoxins, proteins) present
**2.2. Synthetic polymers**				
*PEGMA + PEGDA*	Tram 2020–21	RI 1.335–1.336; G′ 2–10 Pa; shear-thinning; antioxidant release	Mimics vitreous rheology; cataract-protective antioxidants	Limited in vivo duration; antioxidant release mostly early burst
*PVA (γ-ray/STMP crosslinked)*	Yamauchi 1991; Leone 2010	Mesh ≈ 70 nm; G′ 4–1290 Pa; >3 mo degradation	Sterilizable; tunable viscoelasticity	Inflammation; opacification; intraretinal migration (Oliveira 2022)
*PVP (various crosslinkers)*	Hong 1996; Sheikholeslami 2015	Transparency 87–93%; G′ 4–300 Pa; shear-thinning with modifications	Biocompatible in vitro; injectable	Fragmentation → vacuolated macrophages; 50% loss in 4 wks
*PMAGME*	Chirila 1994	Water content 60–90%; injectable	Low fragmentation on injection	Chronic inflammation, retinal disorganization, optic atrophy
**3. In-situ crosslinked hydrogels**				
**3.1. Biopolymers**				
*Oxidized HA + ADH*	Su 2011	RI 1.342–1.344; G′ >1000 Pa; gel in 3 min	Transparent; fast gelation	Too rigid; handling difficult; possible tissue damage
*Vitargus (ABV-1701)*	Chang 2019	In-situ HA gel	Phase II clinical trial; tamponade function	IOP elevation in 3/11 pts; 1 glaucoma surgery
*HA + Chitosan*	Wang 2021	Gelation 1–6 min; transparency 83–96%; ~3 wks degradation	Biocompatible in rats/rabbits	Excess swelling → IOP rise
*HA thiolated (disulfide)*	Yu 2022	Transparency 85–98%; G′ 500–1500 Pa	Biocompatible in vivo	Too rigid; swelling risk
*HA + Silk (enzymatic)*	Raia 2020	RI 1.336; 75–91% transmittance	Potential anti-VEGF delivery	Reduced transparency; oxidative risk (H₂O₂)
*Gellan (Ca²⁺ crosslinked)*	Suri 2006	G′ ≈1000 Pa; 20–30% degradation in 1 wk	Transparent; biocompatible in vitro	Too rigid; short duration
*Chitosan/PVA (thermogel)*	Yu 2020	1–7% polymer; RI 1.33–1.62	Properties close to vitreous at 1%	≥ 3% → non-physiological RI; cataract; IOP rise
**3.2. Synthetic polymers**				
*PEG/PPG/PCL (Vitreogel)*	Liu 2019; Lin 2022	Thermogel 16–20 °C; RI 1.339–1.344; G′ 449 Pa; degradation 3 mo	Functional in rabbits/primates up to 12 mo; proteomic evidence of vitreous-like regeneration	Higher stiffness vs. native vitreous
*PEG thiol/maleimide (8-arm)*	Ran 2021; Wang 2022	Gelation < 1 min; G′ 9.9–26 Pa; RI ~ 1.336; transparency > 80%	Biocompatible 6 mo; shear-thinning	Needs further long-term validation
*Acrylamide gels*	Swindle 2009	Transparency good; adjustable G′	Initial compatibility in rabbits	Residual acrylamide toxicity; long-term safety concerns
*PVA-methacrylate (UV 365 nm)*	Cavalieri 2004	G′ ≈4000 Pa	Clear hydrogel	UV exposure toxic; too rigid
*Peptide SPG-178 (self-assembly)*	Uesugi 2017	Transparency 96.7%; RI 1.3339; G′ 18 Pa	Biocompatible in rabbits; soft gel	Degradation not well defined; short-term data only
*DNA hydrogels (self-assembly)*	Jin 2022	Transparent; shear-thinning; self-healing	Good in vivo tolerance	Excess stiffness; needs optimization

### Effects of silicone oil on imaging

**Fig. 5:**
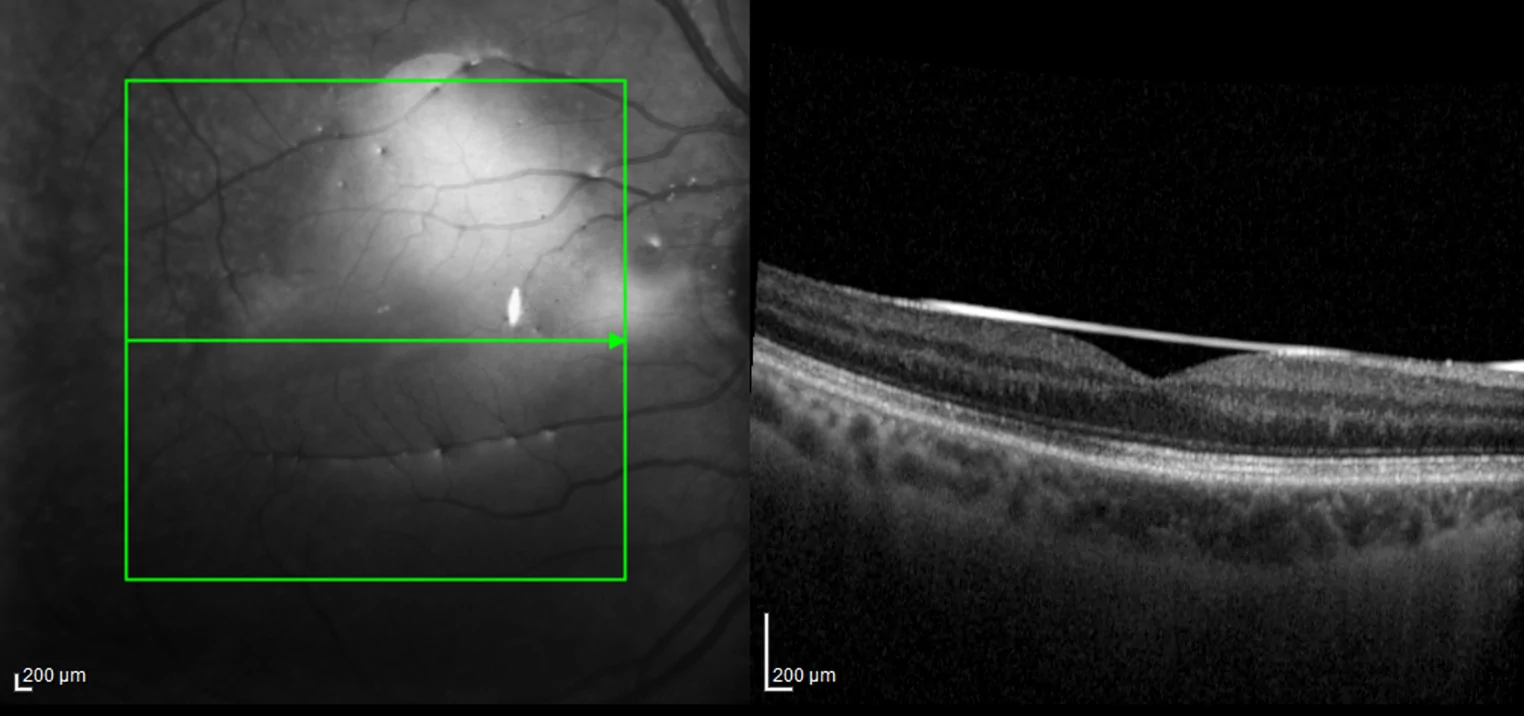
Optical coherence tomography (OCT) of an eye filled with silicone oil tamponade. The macular scan demonstrates a hyperreflective anterior line corresponding to the silicone oil–retina interface, with underlying retinal layers visualized beneath the tamponade agent

On OCT, SO modifies light transmission and reflectivity, causing signal attenuation and reduced visualization of deeper retinal layers, potentially affecting automated segmentation and quantitative assessment of retinal morphology. Clinically, SD-OCT provides a sensitive, non-invasive tool for early detection of silicone oil emulsification, allowing identification of hyperreflective spherical droplets even before clinically evident emulsification on slit-lamp examination. Moreover, recognizing the characteristic epi-, intra-, and subretinal OCT patterns may help guide timely silicone oil removal and closer monitoring of macular involvement, potentially preventing irreversible retinal or optic nerve damage (Fig. 5) [36].

On CT, intraocular SO typically appears hyperdense relative to vitreous, which may be misinterpreted as vitreous hemorrhage or intraocular mass, particularly in non-ophthalmic settings. On MRI, SO shows characteristic signal inversion (hyperintense on T1 and variably hypointense on T2) with possible chemical shift artifacts, potentially complicating the assessment of intraocular or intracranial pathology [83].

#### Impact of silicone oil tamponade on ocular biometry and refractive outcomes

SO induces refractive changes, typically causing a hyperopic shift in phakic and pseudophakic eyes and myopic shifts in aphakia, depending on axial length and oil fill. In SO–filled eyes, swept-source OCT biometry (IOLMaster 700) systematically overestimates axial length (mean ≈ 0.12 mm), potentially leading to hyperopic refractive surprises, particularly in long eyes. Applying the axial length adjustment formula proposed by Zhang et al. (yielding an “adjusted AL”, aAL) effectively neutralizes this bias, reducing the mean AL error to nearly zero and improving IOL power prediction by approximately 0.3 D in eyes longer than 25 mm. Therefore, AL correction using the Zhang formula should be considered before IOL calculation in SO-filled eyes [84]. Accurate IOL power calculation therefore requires specific adjustment formulas and careful preoperative planning.

## Conclusion

Our review shows that silicone oil (SO) remains a crucial tamponade for complex posterior segment surgery. When tailored to pathology and patient factors, SO supports durable anatomical success. Therefore, the choice of SO formulation should be principled: lower-viscosity SOs facilitate handling but emulsify earlier; higher-viscosity SOs better resist emulsification at the cost of more demanding injection/removal; heavy SO is reserved for inferior pathology and selected recurrences. Complication risk increases with dwell time; thereby, many centers plan SOR around 3–6 months, individualizing based on retinal stability, PVR risk, anterior migration, IOP, and keratopathy. Future gains are expected from emulsification-resistant formulations, smarter perioperative planning, including the post-HSO phase, and hydrogel or FCVB platforms that separate tamponade from direct retinal contact. A decision-based approach, considering viscosity, specific gravity, positioning feasibility, and emulsification risk, should guide the individualized selection and timing of SO to maximize safety and visual outcomes.

## Data Availability

No datasets were generated or analysed during the current study.
